# Up-regulation of abscisic acid signaling pathway facilitates aphid xylem absorption and osmoregulation under drought stress

**DOI:** 10.1093/jxb/erv481

**Published:** 2015-11-06

**Authors:** Huijuan Guo, Yucheng Sun, Xinhong Peng, Qinyang Wang, Marvin Harris, Feng Ge

**Affiliations:** ^1^State Key Laboratory of Integrated Management of Pest Insects and Rodents, Institute of Zoology, Chinese Academy of Sciences, Beijing, China; ^2^College of Agronomy, Jiangxi Agricultural University, Nanchang, China; ^3^Department of Entomology, Texas A & M University, College Station, TX 77843, USA

**Keywords:** Abscisic acid, *Acyrthosiphon pisum*, drought, feeding behavior, jasmonic acid, *Medicago truncatula*, osmoregulation, resistance, salicylic acid, xylem absorption.

## Abstract

The activation of the abscisic acid (ABA) signaling pathway reduces water loss from plants challenged by drought stress. The effect of drought-induced ABA signaling on the defense and nutrition allocation of plants is largely unknown. We postulated that these changes can affect herbivorous insects. We studied the effects of drought on different feeding stages of pea aphids in the wild-type A17 of *Medicago truncatula* and ABA signaling pathway mutant *sta-1*. We examined the impact of drought on plant water status, induced plant defense signaling via the abscisic acid (ABA), jasmonic acid (JA), and salicylic acid (SA) pathways, and on the host nutritional quality in terms of leaf free amino acid content. During the penetration phase of aphid feeding, drought decreased epidermis/mesophyll resistance but increased mesophyll/phloem resistance of A17 but not *sta-1* plants. Quantification of transcripts associated with ABA, JA and SA signaling indicated that the drought-induced up-regulation of ABA signaling decreased the SA-dependent defense but increased the JA-dependent defense in A17 plants. During the phloem-feeding phase, drought had little effect on the amino acid concentrations and the associated aphid phloem-feeding parameters in both plant genotypes. In the xylem absorption stage, drought decreased xylem absorption time of aphids in both genotypes because of decreased water potential. Nevertheless, the activation of the ABA signaling pathway increased water-use efficiency of A17 plants by decreasing the stomatal aperture and transpiration rate. In contrast, the water potential of *sta-1* plants (unable to close stomata) was too low to support xylem absorption activity of aphids; the aphids on *sta-1* plants had the highest hemolymph osmolarity and lowest abundance under drought conditions. Taken together this study illustrates the significance of cross-talk between biotic-abiotic signaling pathways in plant-aphid interaction, and reveals the mechanisms leading to alter aphid fecundity in water stresses plants.

## Highlight

Aphid fecundity is curtailed in drought-stressed plants primarily via reduced water status; however, activation of ABA signaling by drought cross-talks with SA and JA signaling to reduce induced plant resistance effects on aphid fecundity.

## Introduction

Drought is a serious agricultural problem because 40% of the world’s agricultural land lies in arid or semi-arid regions (Ehsanpour and Razavizadeh, 2005). Drought harms plants and reduces crop yields by causing cellular water deficits, membrane injury, and reduced enzyme activity (Su *et al.*, 2013). By modifying the quality of host plants, drought can also alter the performance of herbivorous insects. Researchers have predicted that an increase in drought severity could cause outbreaks of pest insects (National Research Council, 2010), as supported by previous cases (Chaves *et al.*, 2003; Turtola *et al.*, 2005; Mody *et al.*, 2009).

Plants challenged by drought have decreased water potential and water content in their leaf tissues (Morgan, 1984). Although plants can avoid excess water loss by reducing stomatal aperture (Schroeder *et al.*, 2001), reducing stomatal aperture suppresses photosynthesis and leads to carbon starvation (Flexas and Medrano, 2002). Drought-induced carbon starvation may result in the re-allocation of primary metabolites into secondary metabolites, which can change host plant nutrition, palatability, and resistance with respect to herbivores (McDowell, 2011). Drought stress, especially in legumes, inhibits biological N fixation and in turn decreases N accumulation in plant tissue (Serraj *et al.*, 1999). Thus, plant responses to water stress, including changes in the plant’s nutritive value, resistance, and water status, should be examined when considering the effect of water stress on plant–insect interactions.

Plant responses to both drought stress and pests, including herbivores and pathogens, involve abscisic acid (ABA) pathway (Lee and Luan, 2012). Drought stress triggers the accumulation of ABA, which induces stomatal closure and decreases transpiration to increase water-use efficiency (Kim and Maik, 2010). The ABA signaling pathway can cross-talk with other phytohormones such as cytokinin, jasmonic acid (JA), and salicylic acid (SA), and such cross-talk can modify how plants interact with herbivorous insects (Mauch-Mani and Mauch, 2005; Ding and Oldroyd, 2009; Fan *et al.*, 2009). ABA typically contributes to plant resistance to biotic invasion by suppressing SA-dependent defenses and by up-regulating JA-dependent defenses, as well as suppressing the synthesis of some secondary metabolites, such as indole glucosinolate in Arabidopsis (Mauch-Mani and Mauch, 2005; Mosher *et al.*, 2010; Hillwig *et al.*, 2015). Furthermore, the ABA signaling pathway in legumes can regulate cytokinin induction during nodulation and thereby affect N fixation and N assimilation (Ding *et al.*, 2008; Tominaga *et al.*, 2009). Thus, ABA integrates and fine-tunes both abiotic and biotic stress response signaling networks. The effects of ABA on plant water status, resistance, and nutrition also warrant examination in the context of plant–insect interactions.

Previous reports indicate that some aphids have greater reproductive capacity under drought conditions than under non-drought conditions (Jactel *et al.*, 2012; Mewis *et al.*, 2012). The most accepted explanation for this phenomenon was proposed by White *et al*. (1969), who suggested that drought increases the hydroxylation of proteins, which subsequently increases the levels of free amino acids available to aphids. Before their stylets reach the phloem sap to access these free amino acids, however, the aphids must overcome a variety of other induced defenses, including those that are located in the epidermis and the mesophyll (Smith and Boyko, 2007; Guo *et al.*, 2014). The activation of the ABA signaling pathway can regulate the defensive phytohormones SA and JA under drought stress, which can affect the penetration phase of aphid feeding. Furthermore, once an aphid has established feeding sites in phloem sieve elements, the water potential of the host plant can affect the xylem absorption phase; the latter phase helps aphids deal with the osmotic pressure of the phloem sap and therefore affects the duration of phloem feeding (Huberty and Denno, 2004; Pompon *et al*., 2010, 2011). The plant–aphid interaction is a dynamic process involving different aspects of plant quality interfacing with each feeding phase of the aphid to ameliorate or exacerbate the interaction on the way to determining net effects on the aphid.

In the current study, we used the pea aphid, *Acyrthosiphon pisum*, and *sta-1*, an ABA-insensitive mutant of *Medicago truncatula*, and its wild-type control A17 to examine the effects of drought-induced ABA signaling on aphid feeding behavior. Our specific goals were: (i) to determine the differences in nutrition, resistance, and water status between two plant genotypes that differed in the ABA signaling pathway; and (ii) to determine the effect of these changes on the different feeding stages of aphids.

## Materials and methods

### Host plants

The *M. truncatula* sensitivity-to-ABA mutant (*sta-1*) and an isogenic wild-type plant (cv. A17) were kindly provided by Professor Oldroyd, Department of Disease and Stress Biology, John Innes Centre, UK. *sta-1* is insensitive to ABA for lateral root initiation and stomatal closure (Ding *et al.*, 2008). The plants were pre-treated in KCl-MES buffer (50mM KCl and 10mM MES, pH 6.12) under continuous white light (250 µmol m^–2^ s^–1^ for 90min to ensure maximal stomatal opening) and then treated with buffer alone or with buffer containing 25 µM ABA. With ABA treatment, all stomata close in the wild-type leaves but all stomata remain open in the *sta-1* mutant leaves. *sta-1* also shows defects in plant growth and seed germination (Ding *et al.*, 2008).

*Medicago truncatula* A17 and *sta-1* plants were germinated and inoculated with *Sinorhizobium meliloti* 1021 as described previously (Guo *et al.*, 2013). After they had grown in sterilized soil for 2 weeks, the *M. truncatula* seedlings were individually transplanted into plastic pots (35cm diameter and 28cm height) containing sterilized loamy field soil (organic carbon 75g kg^–1^, N 500mg kg^–1^, P 200mg kg^–1^, K 300mg kg^–1^) and placed in the greenhouse with natural sunlight and temperatures ranging from 16 °C to 30 °C at the Observation Station of the Global Change Biology Group, Institute of Zoology, Chinese Academy of Science in Xiaotangshan County, Beijing, China (40°11'N, 116°24'E). Each genotype contained 160 plants and there were 320 plants in total.

Forty-day-old *M. truncatula* plants were assigned to two irrigation regimes (treatments): 3000ml of water per week (well-watered) and 600ml per week (water-stressed). Soil water potentials in well-watered and water-stressed treatments were determined to be approximately –0.08MPa and –0.45MPa (Soil Moisture Equipment, Santa Barbara, CA, USA). Each pot was placed on a 10cm deep plastic tray (40cm diameter) to retain drainage water. After the plants had grown for 7 weeks, they were used for the four groups of assays described in the following sections.

### Aphid infestation

The pea aphid *A. pisum* was originally collected from *Pisum sativum* L. at Yunnan Province and was reared in the laboratory for 5 years on *Vicia faba* with 14h light (25 °C)/10h dark (22 °C) in photoclimatic chambers (Safe PRX-450C, Ningbo, China).

Sixteen plants of each genotype and each water treatment (64 plants in total) were selected for aphid infestation. Each plant was infested with aphids by placing a total of 50 apterous fourth instar nymphs on the fourth and fifth trifoliate leaves (counting from the base), and the leaves, which were terminal and mature, were caged (80 mesh gauze). Another 16 plants of each genotype and each water treatment (64 plants in total) were selected as control plants, and their corresponding leaves were caged in the same way but without aphids. After they were infested for 24h, eight plants of each genotype and water treatment were selected to measure leaf water potential using the PSYPRO water potential system. The same plants were later used to measure relative leaf water content. To determine phytohormone content and the relative expression of genes in the ABA, JA, and SA signaling pathways, another eight infested and uninfested plants of each combination of water treatment and genotype were selected, and 500mg of leaves from each plant were harvested separately after 24h of aphid infestation; the leaf samples were immediately stored in liquid N to measure phytohormones and defensive gene expression. As described later in the Materials and methods, leaf N concentration and amino acid concentration in phloem were determined on each of eight control plants of each genotype and each water treatment (32 plants in total).

### Aphid feeding behavior as affected by drought stress and host plant genotype

Twenty-four plants from each combination of water treatment and genotype were randomly selected (96 plants in total). After each plant was infested with one apterous adult, aphid feeding behavior was recorded for 12h using the electrical penetration graph (EPG) method (Guo *et al.*, 2014). Waveform patterns were scored according to the categories described by Tjallingii and Esch (1993): non-penetration (NP); pooled pathway phase activities (C); salivary secretion into sieve elements (E1); phloem ingestion (E2); derailed stylets (F); and xylem ingestion (G). According to Alvarez *et al.* (2006), four EPG parameters were selected as indicators of induced resistance to aphids: (i) the minimum duration of waveform C within a probe before E1; (ii) the number of probes shorter than 3min (test probes) that occur before the first E1 wave, which probably reflect the role of epidermis/mesophyll resistance; (iii) the duration to the first E1; and (iv) the duration to the first E2, which indicates the ease of phloem access and acceptance. Two EPG parameters were selected as indicators of aphid xylem activity: (i) the duration to the first G wave; and (ii) the average duration of G periods. The average durations of E2 periods (total time spent in E2) were selected as indicators of phloem suitability as well as general plant suitability.

### Aphid population abundance, water content, and hemolymph osmolarity as affected by drought stress and host genotype

Twelve plants from each combination of water treatment and genotype (48 plants in total) were randomly selected, and five apterous fourth instar nymphs were caged on each plant using 80 mesh gauze. The nymphs were allowed to develop into adults and reproduce on each plant for 23 d. Aphid numbers on each plant were determined on day 7, 11, 15, 19, and 23. At the end of the experiment, the aphids were brushed from each plant and collected, and 50 adults from each plant were analyzed for hemolymph osmolarity. Aphid hemolymph was collected according to Shakesby *et al.* (2009). The osmolarity of 10 μl samples was determined using a pressure osmometer 5220 (Wescor Inc., Logan, UT, USA). In addition, 35 adults from each plant were weighed immediately after they were collected, dried at 60 °C for 24h, and then weighed again. Water content was determined by subtracting the dry weight from the fresh weight of each aphid.

### Plant stomatal conductance and water transpiration rate

Stomatal conductance, photosynthetic rate, and water transpiration rate of uninfested plants were measured on the fourth to eighth terminal mature trifoliate leaves from the base of the shoot with a Li-Cor 6400 gas exchange system (6400-40; Li-Cor Inc., Lincoln, NE, USA) between 07:30h and 10:30h. The incoming CO_2_ concentration was adjusted to 400 μmol mol^–1^. Relative humidity corresponded to ambient conditions (55–60%). Before gas exchange was measured, illumination was set to 90% red and 10% blue, the temperature was set to 25 °C, and photosynthetically active radiation (PAR) for the leaf in the measuring cuvette was 1200 μmol m^–2^ s^–1^. Measurements were taken when the CO_2_ assimilation rate was stable for at least 2min. The water-use efficiency was measured using the formula: water-use efficiency (μmol/mmol)=photosynthetic rate/water transpiration rate (Donovan *et al.*, 2007; Des Marais *et al.*, 2014).

### Plant water potential and relative water content

Three leaves per plant were selected to measure leaf water potential with the PSYPRO water potential system (Wescor Inc.). Leaf relative water content (RWC) was measured using the formula: RWC (%)=(FW–DW)×100/(TW–DW) (Barrs and Weatherley, 1962). Fresh weight (FW) was measured and leaves were left to rehydrate in distilled water for 24h at 15 °C in darkness to obtain the weight at full turgor (TW). Leaf dry weight (DW) was measured after 72h at 65 °C.

### Plant N concentration and leaf total amino acid concentration

The above-ground leaves from each plant were collected and oven-dried (65 °C) for 72h. We then ground 0.3g and 0.1g of the leaves of each plant to a fine powder (0.85mm sieve) and analyzed these samples for N concentration and total free amino acid in leaves. N concentration in leaves was determined using Kjeltec N analysis (Foss automated Kjeltec™ instruments, Model 2100, Hillerød, Denmark). Roots of each plant were carefully removed from the soil and washed. A stereomicroscope aided counting the nodules on the entire root system of each plant. The total free amino acid in leaves was measured using the methods of Godzik and Linskens (1970).

### Plant phloem amino acid concentrations

For quantifying amino acid concentrations in phloem, phloem exudates were obtained from three trifoliate per plant by using the EDTA exudation technique of Douglas (1993). Twenty individual amino acids per host plant were determined, namely alanine, arginine, asparagine, aspartate, cysteine, glutamate, glutamine, glycine, histidine, isoleucine, leucine, lysine, methionine, phenylalanine, proline, serine, tyrosine, threonine, tryptophan, and valine. The amino acids in each sample were analyzed by reverse-phase HPLC with pre-column derivatization using *o*-phthaldialdehyde (OPA) and 9-fluorenylmethyloxycarbonyl (FMOC). Amino acids were quantified by comparison with the AA-S-17 (Agilent, PN: 5061-3331) reference amino acid mixture, supplemented with asparagine, glutamine, and tryptophan (Sigma-Aldrich Co., St. Louis, MO, USA). Standard solutions were prepared from a stock solution by diluting with 0.1M HCl. Free amino acid concentrations of the five standard solutions are 250, 100, 50, 25, and 10 pmol μl^–1^. Before injection in the HPLC, 10 μl of amino acid sample, 20 μl of sodium borate buffer (0.4 N, pH 10.4), 10 μl of OPA, 10 μl of FMOC, and 50 μl of water were mixed. The analysis was performed using Agilent 1100 HPLC systems (Agilent Technologies, Palo Alto, CA, USA). A reverse phase Agilent Zorbax Eclipse C18 column AAA (5 μm, 250 mm×4.6mm) were used for the chromatographic separation with a fluorescence detector. The column was maintained at 35 °C with a gradient (1ml min^–1^ flow) programmed as follows: 98/2 (1min) to 43/57 (25min) to 0/100 (34min) to 98/2 (42min hold) of eluent A/eluent B. Eluent A was 40mM disodium phenyl phosphate buffer (pH 7.8 adjusted with sodium hydroxide). Eluent B was a 45% acetonitrile, 45% methanol, and 10% water. Chemstation Plus Family for LC software was used for data acquisition and analysis. Amino acid concentrations were quantified by comparison of sample peak areas with standard curves of 20 reference amino acids (Agilent Chemical Co.).

### Measurement of phytohormones

Approximately 300mg of fresh plant leaves was homogenized with liquid nitrogen. A 1.5ml aliquot of extraction buffer (2:1:0.005, isopropanol:water:concentrated HCl) was added to each sample. Samples were agitated for 30min at 4 °C. 1.5ml of CH_2_Cl_2_ was added, followed by agitation for another 30min and then centrifugation at 13 000 *g* for 5min. After centrifugation, two phases were formed and plant debris was in the middle of two layers. The aqueous phase was discarded and the lower layer collected and concentrated in a rotary evaporator, and re-solubilized in 200 μl of 60% MeOH. JA, SA, ABA, and *cis*(+)-12-oxophytodienoic acid (OPDA) concentrations were quantified by comparison of sample peak areas with standard curves of reference phytohormones (Agilent Chemical Co.).

A Perkin-Elmer 200 liquid chromatograph coupled with an Analytical Biosystems Sciex API 4000 mass spectrometer, with triple quadrupole and turbo spray ion source, was used. Mass spectrometric experimental conditions were as follows: Q2 gas pressure, 3.7×10^−5^ Torr; Q1 and Q3 resolution, 0.7 amu; cycle time, 605ms (11 transitions with a dwell time of 50ms); spray voltage, 5.5kV; sheath gas flow rate, 55ml min^–1^; auxiliary gas flow rate, 20ml min^–1^; auxiliary gas temperature, 400 °C. Air was used as sheath and auxiliary gas. A Symmetry Waters C18 column (2.1×50mm, 5 μm particle diameter) was used, and gradient chromatographic separation was performed at a flow rate of 0.2ml min^–1^ as follows: 5% (1min) to 95% (5min) to 95% (7min) to 5% (7.1min) to 5% (15min) of eluent A/eluent B. Eluent A was 0.1% HCOOH. Eluent B was 100% acetonitrile. The injected volume was 10 μl.

### Expression of genes associated with induced resistance as determined by quantitative RT-PCR

The RNA Easy Mini Kit (Qiagen) was used to isolate total RNA from *M. truncatula* leaves, and 1 μg of RNA was used to synthesize cDNAs. mRNAs of the following target genes used as markers of phytohormone signaling were quantified by real-time quantitative PCR: pathogenesis-related protein (*PR*), β-1,3-glucanase (*BGL*), endochitinase (*CHTN*), 12-OPDA reductase (*OPR*), cysteine proteinase inhibitor (*PI*), and ABA-responsive protein (*ABR*). *PR*, *BGL*, and *CHTN* are in the SA signaling pathway, *LOX* and *PI* are in the JA signaling pathway, and *ABR* is in the ABA signaling pathway (Iuchi *et al.*, 2001; Colditz *et al.*, 2004; Guo *et al.*, 2014). Specific primers for genes were designed from the *M. truncatula* expressed sequence tag (EST) sequences using PRIMER5 software (see Supplementary Table S1 available at *JXB* online). The PCRs were performed in 20 μl reaction volumes that included 10 μl of 2×SYBRs Premix EX Taq™ (Qiagen) master mix, 5mM of each gene-specific primer, and 1 μl of cDNA template. Reactions were carried out on the Mx 3500P detection system (Stratagene) as follows: 2min at 94 ºC; followed by 40 cycles of 20s at 95 ºC, 30s at 56 ºC, and 20s at 68 ºC; and finally one cycle of 30s at 95 ºC, 30s at 56 ºC, and 30s at 95 ºC. The melting curves were used to determine the specificity of the PCR products. A standard curve was derived from the serial dilution to quantify the copy numbers of target mRNAs. The housekeeping gene *β-actin* was used as the internal quantitaive PCR standard to analyze plant gene expression. The relative level of each target gene was standardized by comparing the copy numbers of target mRNA with copy numbers of *β-actin*, which is believed to remain constant under different treatment conditions. The levels of *β-actin* transcripts in the control were examined in every PCR plate to eliminate systematic error. The fold changes of the target genes were calculated using the 2^–ΔΔCt^ normalization method. Each combination of aphid infestation, plant genotype, and CO_2_ level was represented by four biological replicates, and each biological replicate contained four technical repeats.

### Statistical analyses

Three-way ANOVAs were used to compare uninfested and infested plants for ABA, JA, OPDA, and SA content, ABA-, JA-, and SA-related gene expression, plant transpiration rate, plant water-use efficiency, water potential, and relative water content. Two-way ANOVAs was used to analyze plant N concentration, individual amino acids in plant phloem, aphid feeding behavior, aphid osmolarity, and aphid water content. Tukey’s multiple range tests were used to separate means when ANOVAs were significant. Repeated measures ANOVAs were used to compare aphid numbers. All data were checked for normality and equality of residual error variances and were appropriately transformed (log or square-root) if needed to satisfy the assumptions of analysis of variance.

## Results

### Aphid population abundance and feeding behavior

**Fig. 1.**
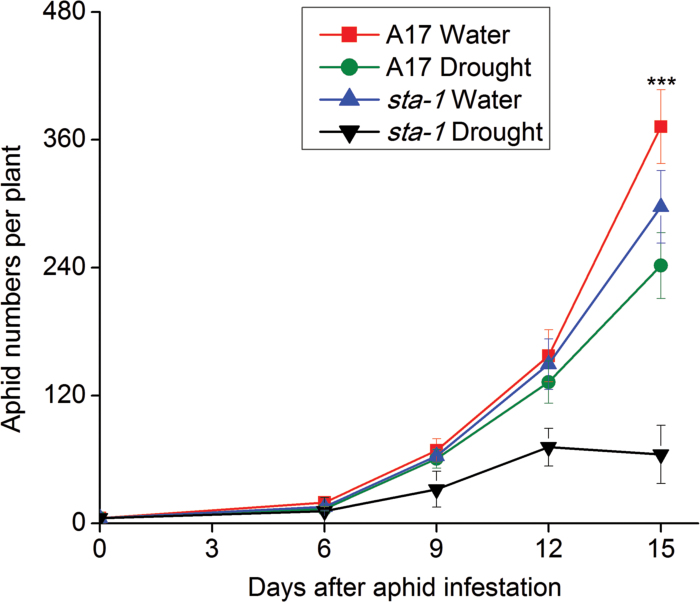
Numbers of pea aphids per plant when fed on wild-type A17 and *sta-1* (sensitivity-to-ABA) mutant plants grown under well-watered (water) and drought-stressed (drought) conditions. Each value is the mean (±SE) of 12 replicates. Significant differences at *P*<0.05 are indicated by asterisks. (This figure is available at *JXB* online.)

Relative to a well-watered condition, the abundance of pea aphids associated with A17 and *sta-1* was decreased by 35.1% and 78.2%, respectively, under drought stress beginning 15 d post-infestation. Moreover, pea aphids reared on *sta-1* plants exhibit a 20.3% and 73.2% decrease in population growth compared with those reared on A17 plants under well-watered and drought-stressed conditions, respectively (Fig. 1).

**Fig. 2.**
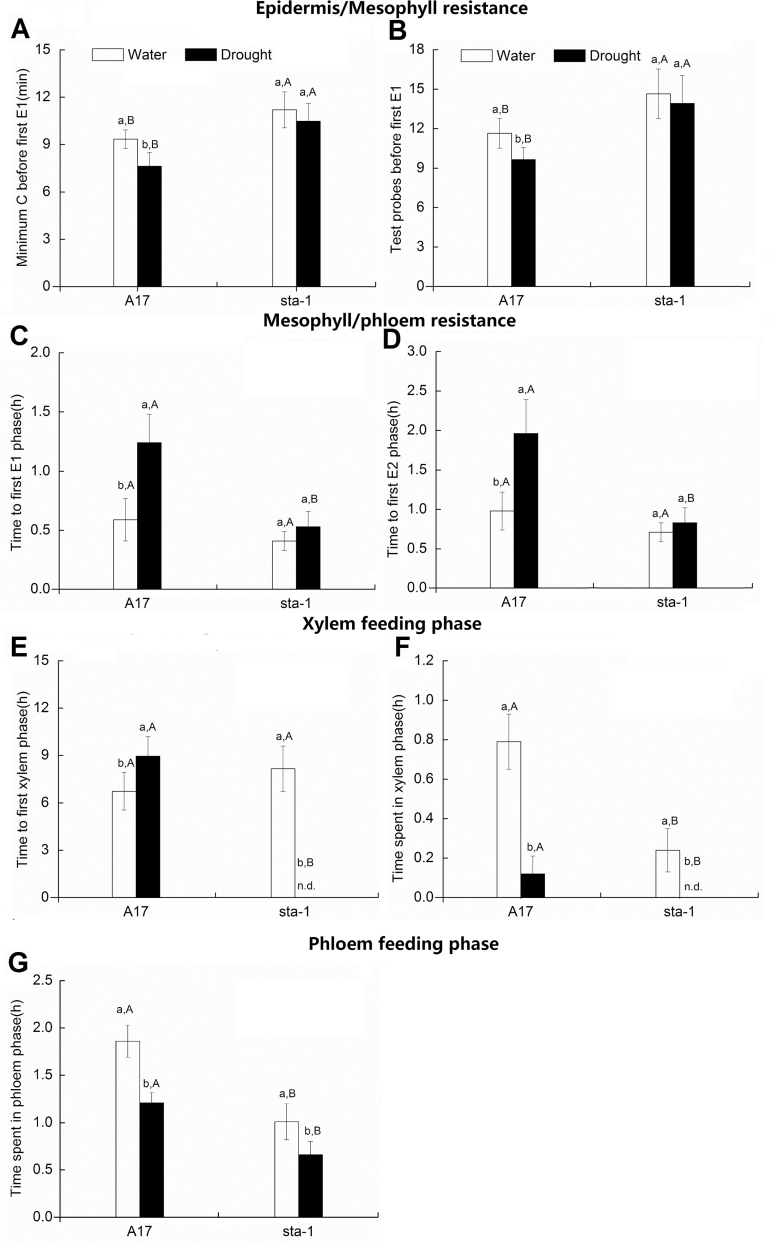
Electrical penetration graph (EPG) results for pea aphids feeding for 12h on A17 and the *sta-1* mutant plants grown under well-watered (water) or drought conditions. (A) Minimum C before E1; (B) number of probes <3min before first E1; (C) time to first E1; (D) time to first E2; (E) time to first G phase; (F) time spent in the xylem phase; and (G) time spent in the phloem phase. Each value is the mean (±SE) of 24 biological replicates. Different lower case letters indicate significant differences between water treatments within the same genotype. Different upper case letters indicate significant differences between genotypes within the same water treatment as determined by Tukey’s multiple range test at *P*<0.05.

The EPG data were used to indicate whether the aphids encountered epidermis/mesophyll resistance or mesophyll/phloem resistance. With respect to epidermis/mesophyll resistance, drought stress shortened the minimum duration of C and decreased the number of probes <3min before E1 for aphids on A17 plants but not for aphids on *sta-1* plants (Fig. 2A, B). Aphids encountered more epidermis/mesophyll resistance on *sta-1* plants than on A17 plants regardless of drought treatment (Fig. 2A, B).

With respect to mesophyll/phloem resistance and phloem resistance, drought stress prolonged the time to the first E1 and the time to the first E2 for aphids on A17 plants but not for aphids on *sta-1* plants (Fig. 2C, D). Under well-watered conditions, the time before the first E1 and E2 was shorter on A17 than on *sta-1* plants, and drought stress increased the difference in mesophyll/phloem resistance between the genotypes (Fig. 2C, D).

Regardless of plant genotype, drought stress significantly prolonged the time to the first xylem phase and decreased the time spent in the xylem phase (Fig. 2E, F). Furthermore, aphids spent a longer time in the xylem phase on A17 plants than on *sta-1* plants (Fig. 2E, F). Under drought conditions, the xylem phase of feeding was undetectable for aphids on *sta-1* plants (Fig. 2F).

Regardless of plant genotype, drought stress significantly decreased the phloem feeding time. Moreover, the duration of the phloem phase was greater for aphids on A17 plants than on *sta-1* plants (Fig. 2G).

### ABA signaling pathway was affected by drought and aphid infestation

**Fig. 3.**
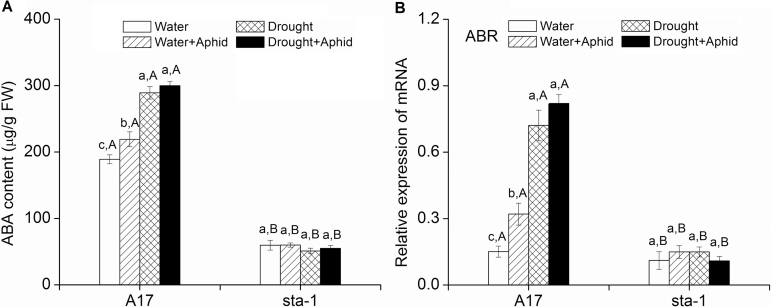
ABA content and relative expression of the downstream ABA-responsive protein gene (*ABR*) for two *M. truncatula* genotypes grown under well-watered (water) or drought conditions with and without pea aphid infestation. Each value represents the mean (±SE) of four replicates. Different lower case letters indicate significant differences among the combinations of aphid treatment and water treatment within the same genotype. Different upper case letters indicate significant differences between genotypes within the same water treatment and aphid treatment as determined by Tukey’s multiple range test at *P*<0.05.

To determine whether drought stress and aphid infestation regulate the ABA signaling pathway, we examined the ABA content and the downstream gene *ABR* in both A17 and *sta-1* plants (see Supplementary Table S2 at *JXB* online). Drought stress up-regulated ABA content and expression of *ABR* in A17 plants while having little effect on the ABA signaling pathway in *sta-1* plants regardless of the status of infestation by aphids (Fig. 3). Furthermore, aphid infestation increased the ABA content and expression of *ABR* in A17 plants under the well-watered conditions but did not affect them under drought stress (Fig. 3).

### Phytohormone-dependent defense induced by pea aphid

**Fig. 4.**
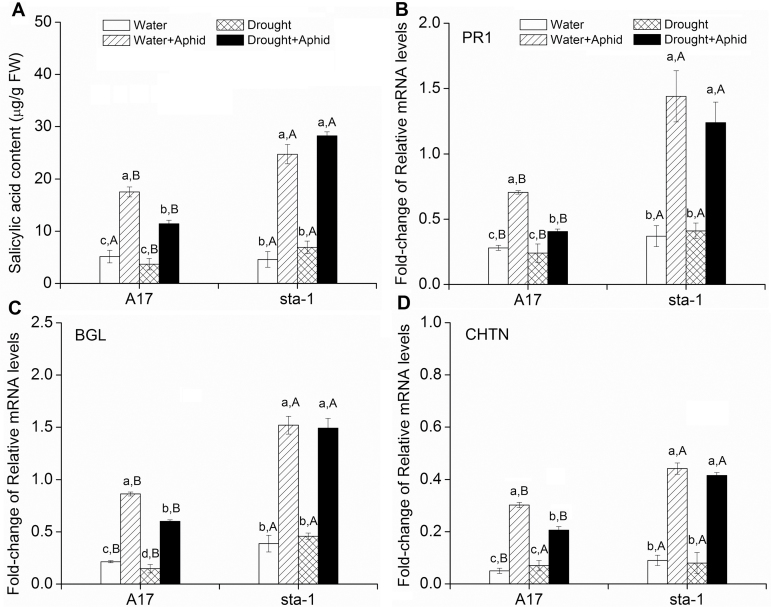
SA content and expression of genes involved in the SA signaling pathway in two *Medicago truncatula* genotypes grown under well-watered or drought conditions with and without pea aphid infestation. (a) SA content; (b) pathogenesis-related protein (*PR*); (c) β-1,3-glucanase (*BGL*); and (d) endochitinase (*CHTN*) expression. Each value is the mean (±SE) of eight replicates. Different lower case letters indicate significant differences among the combinations of aphid treatment and water treatment within the same genotype. Different upper case letters indicate significant differences between genotypes within the same water treatment and aphid treatment as determined by Tukey’s multiple range test at *P*<0.05.

To investigate the induced defenses of *M. truncatula* plants that are drought stressed and infested by aphids, we assessed the SA and JA signaling pathway-dependent defenses. A comparison between uninfested and infested plants of both plant genotypes indicated that aphid infestation up-regulated the expression of SA pathway-related genes including *BGL*, *PR*, and *CHTN* regardless of water treatment (Fig. 4; Supplementary Table S2 at *JXB* online). When plants were infested by aphids, drought down-regulated the SA content and expression of the downstream key genes *BGL*, *PR*, and *CHTN* in A17 plants but did not affect the expression of SA-dependent genes in *sta-1* plants (Fig. 4). Regardless of drought treatment, the expression of genes involved in the SA signaling pathway of A17 plants was lower than that of *sta-1* plants when infested by aphids (Fig. 4).

**Fig. 5.**
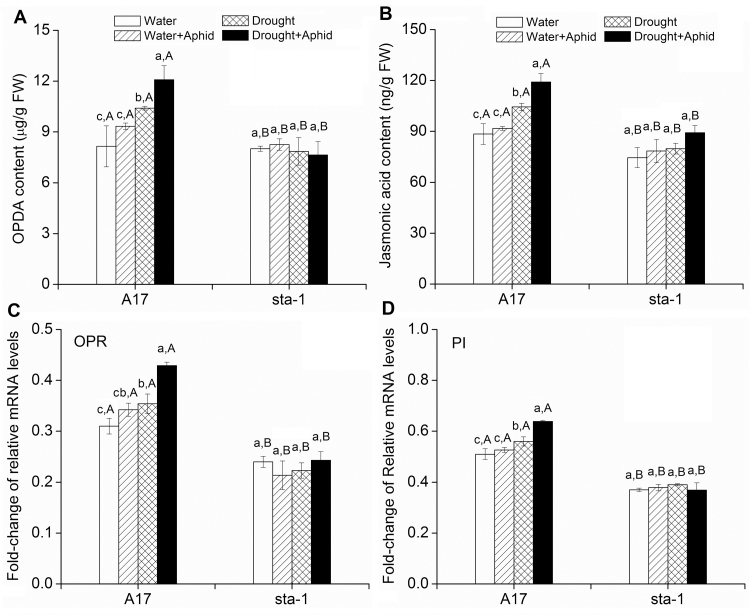
Key metabolites and relative expression of key genes involved in the JA signaling pathway for two *M. truncatula* genotypes grown under well-watered or drought conditions with and without pea aphid infestation. (A) (+)-12-oxophytodienoic acid (OPDA) content; (b) JA content; (c) 12-OPDA reductase (*OPR*) expression; and (d) cysteine proteinase inhibitor (*PI*) expression. Each value represents the mean (±SE) of eight replicates. Different lower case letters indicate significant differences among the combinations of aphid treatment and water treatment within the same genotype. Different upper case letters indicate significant differences between genotypes within the same water treatment and aphid treatment as determined by Tukey’s multiple range test at *P*<0.05.

Drought stress significantly increased the JA signaling pathway in terms of OPDA and JA contents and the expression of OPR and PI in A17 plants regardless of aphid infestation status (Fig. 5; Supplementary Table S2 at *JXB* online). In *sta-1* plants, drought stress decreased the OPDA content but did not affect the JA content or the expression of genes in the JA signaling pathway (Fig. 5). Aphid infestation did not greatly affect the OPDA and JA content or expression of *OPR* and *PI* in either genotype under well-watered conditions (Fig. 5). With drought stress, however, aphid infestation increased OPDA and JA content and up-regulated *OPR* and *PI* expression in A17 plants, and did not affect the JA signaling pathway in *sta-1* plants. Regardless of drought treatment, the expression of genes involved in the JA signaling pathway of A17 plants was higher than that of *sta-1* plants when infested by aphids (Fig. 5).

### Nitrogen and amino acid concentration in plants

**Fig. 6.**
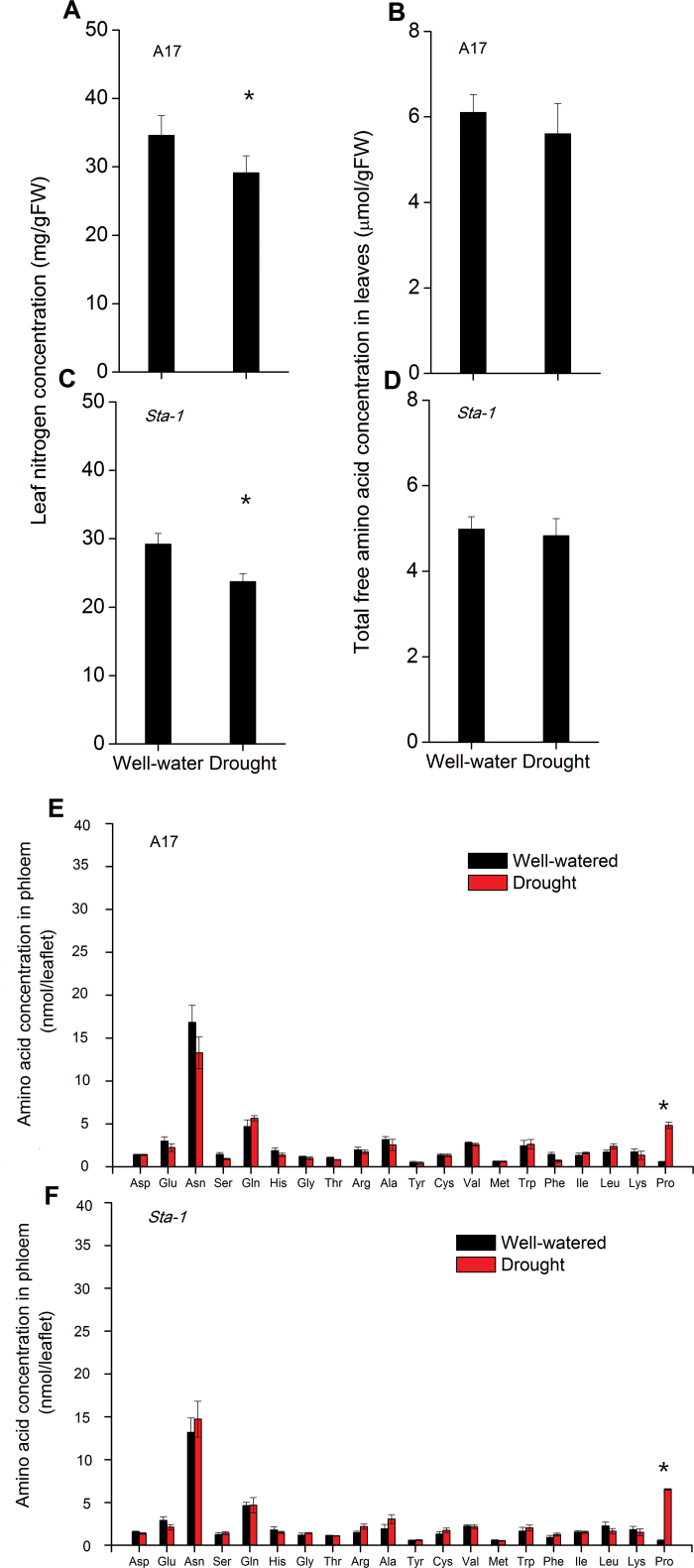
N concentration and phloem amino acid concentration for two *M. truncatula* genotypes grown under well-watered or drought conditions without pea aphid infestation. Significant differences at *P*<0.05 are indicated by asterisks.

We also examined the N concentration, total amino acid concentration of leaves, as well as individual amino acids in phloem of A17 and *sta-1* plants (Fig. 6). Drought decreased the leaf N concentration but did not affect leaf total amino acid concentration in both A17 and *sta-1* plants (Fig. 6A–D). Moreover, drought stress did not significantly affect the concentrations of individual amino acid in the phloem of A17 and *sta-1* plants except for that of proline (Fig. 6E, F). Drought stress significantly increased proline in both genotypes (Fig. 6E, F).

### Water status of plants and aphids

**Fig. 7.**
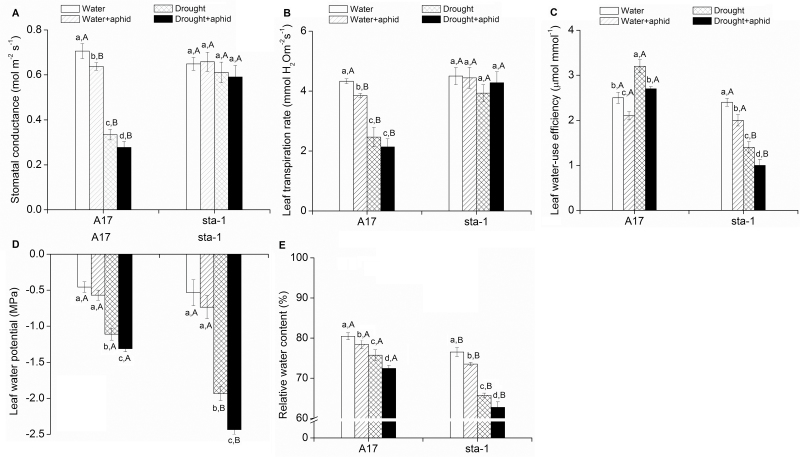
Gas exchange parameters and water status in two *Medicago truncatula* genotypes grown under well-watered and drought conditions with and without pea aphid infestation. (A) Stomatal conductance; (B) transpiration rate; (C) water-use efficiency (D) water potential; and (E) relative water content. Each value represents the mean (±SE) of eight replicates. Different lower case letters indicate significant differences among the combinations of aphid treatment and water treatment within the same genotype. Different upper case letters indicate significant differences between genotypes within the same water treatment and aphid treatment as determined by Tukey’s multiple range test at *P*<0.05.

Drought stress significantly decreased stomatal aperture and the transpiration rate of A17 plants but did not affect those of *sta-1* plants regardless of aphid status (Fig. 7A, B; Supplementary Table S3 at *JXB* online). Under drought stress, A17 plants had a lower stomatal conductance and transpiration rate than *sta-1* plants (Fig. 7A, B).

To test whether the decreased transpiration rate improved plant water status under drought stress, we determined the plant water-use efficiency, water potential, and relative water content of both genotypes (see Supplementary Table S3 at *JXB* online). Drought significantly increased the leaf water-use efficiency of A17 plants but decreased that of *sta-1* plants regardless of the aphid infestation status (Fig. 7C). In response to drought stress, the water potential of A17 plants and *sta-1* plants was decreased 1.4- and 2.6-fold for uninfested plants, and 1.3- and 2.3-fold for aphid-infested plants (Fig. 7D). The relative water content of A17 and *sta-1* plants was decreased 5.8% and 14.1% for uninfested plants, and 7.6% and 14.7% for aphid-infested plants in response to drought stress (Fig. 7E). A17 plants had much higher water-use efficiency, water potential, and water content than *sta-1* plants under drought stress (Fig. 7C, D).

**Fig. 8.**
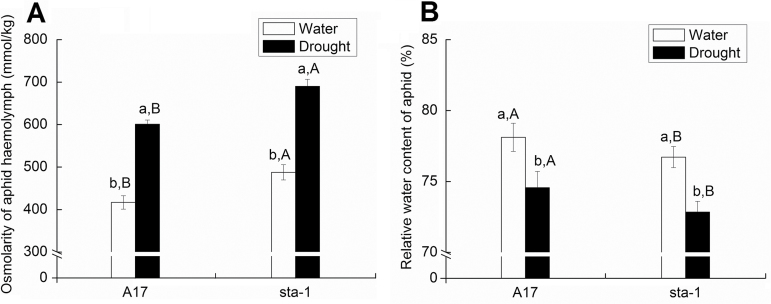
Hemolymph osmolarity and relative water content of pea aphids when fed on wild-type A17 and the *sta-1* (sensitivity-to-ABA) mutant plants grown under well-watered (water) and drought stress conditions. (A) Hemolymph osmolarity; (B) relative water content. Different lower case letters indicate significant differences between water treatments within the same genotype. Different upper case letters indicate significant differences between genotypes within the same water treatment as determined by Tukey’s multiple range test at *P*<0.05.

Relative to well-watered conditions, hemolymph osmolarity of pea aphids reared on A17 and *sta-1* plants was increased by 20.2% and 41.6% under drought stress. The water content of pea aphids reared on A17 and *sta-1* plants was increased by 32.4% and 50.6% under drought stress. Hemolymph osmolarity was higher and water content was lower for aphids on *sta-1* plants than for those on A17 plants (Fig. 8; Supplementary Table S4 at *JXB* online).

## Discussion

Up-regulation of the ABA signaling pathway is an important and well-studied characteristic of plants subjected to drought stress (Kuromori *et al.*, 2014). ABA is a stress signal and also required to fine-tune growth and development under non-stress conditions. The physiological roles of ABA include regulation of nutrition allocation, stomatal aperture, hydraulic conductivity, as well as defensive metabolism in plants (Raghavendra *et al.*, 2010). Here, we report that the up-regulated ABA signaling pathway decreased epidermis/mesophyll resistance but increased mesophyll/phloem resistance of A17 plants against aphids under drought conditions. Furthermore, the function of ABA to close stomata could significantly increase the water-use efficiency of A17 plants on which pea aphids can still conduct their xylem feeding under drought conditions. In contrast, when the ABA signaling pathway was impaired under drought, the aphid has difficulty in absorbing water from the xylem in *sta-1* plants, which dramatically decreased their feeding efficiency and population abundance.

**Fig. 9.**
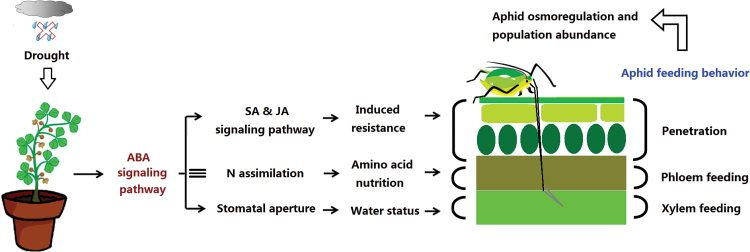
A model summarizing the effects of the up-regulated ABA signaling pathway on aphid feeding in the *M. truncatula*–pea aphid system under drought stress. ≡indicates that drought-induced up-regulation of the ABA signaling pathway did not affect the N nutrition of plants.

The responses of sap-sucking aphids to drought have been broadly reviewed and still lack consensus. Koricheva *et al.* (1998) argued that sap suckers performed better on water-stressed plants than on non-stressed plants, whereas Huberty and Denno (2004) concluded the opposite. To explain this conflict, researchers have presented three hypotheses: the plant-stress hypothesis, the pulsed-stress hypothesis, and the plant-vigor hypothesis (White, 1974; Price, 1991; Huberty and Denno, 2004). The plant-stress hypothesis, which has so far attracted the most attention, asserts that drought increases the hydroxylation of proteins, which subsequently increases the level of free amino acids that enhance insect growth and reproduction (White 1974). Notably, only some field experiments support the notion that aphid density increases on water-stressed plants; the experimentally imposed water stress, however, often negatively affects aphid performance (Huberty and Denno, 2004). The enhanced performance of aphids in natural situations may be due to their suffering intermittent water stress with intervening recovery. The periods of water recovery may allow aphids to benefit from stress-induced increases in plant nitrogen. In the current study, the host plants continuously suffered from water stress which negatively affected the population abundance of the aphid (Fig. 1). Moreover, as shown in Fig. 9, plant resistance ability, amino acid nutrition, and water status are all known to be capable of affecting certain feeding stages of aphids (Alvarez *et al.*, 2006; Nalam *et al.*, 2012; Sun *et al.*, 2015). In the following sections, we will discuss the effect of drought on resistance, nutritive value, and the water status of plants and their interactions with different stages of aphid feeding.

### Aphid penetration stage

Induced resistance is measured by the elapsed time that aphids spend between arriving on a leaf and their first feeding on phloem. Plant defenses against aphids are co-ordinated by several interacting signaling systems and especially by the JA and SA signaling pathways (Felton and Korth, 2000). These signaling pathways are interconnected in a complex network, which appears to be regulated by the ABA signaling pathway (Adie *et al.*, 2007; Asselbergh *et al*., 2007, 2008). Experiments with exogenous application of ABA showed that enhanced ABA levels correlated with an increased JA signaling pathway and a reduction of the SA signaling pathway (Mauch-Mani and Mauch, 2005). Moreover, experiments in Arabidopsis with the use of ABA-deficient mutants showed that a deficiency in the ABA signaling pathway increased the plant resistance against *Myzus persicae*, and decreased the population abundance of the aphid (Kerchev *et al.*, 2013; Hillwig *et al.*, 2015). Similarly in *M. truncatula*, the population abundance of aphids reared on ABA-insensitive mutant *sta-1* plants was lower than that of those on A17 plants, which may be partially due to the increased epidermis/mesophyll resistance in *sta-1* plants (Figs 1, 2A, B). Our previous study found that the SA signaling pathway of *M. truncatula* was implicated in the epidermis/mesophyll resistance against pea aphids (Guo *et al.*, 2014). Higher expression of the SA signaling pathway in *sta-1* plants prolonged the duration of the C wave and increased the number of test probes before the E1 phase (Fig. 2A, B). Additionally, JA signaling was considered as an effective resistance in Arabidopsis against *M. persicae* (Ellis *et al.*, 2002). In *M. truncatula*, the activation of the JA signaling pathway is important for resistant genotype (Jester) expression against blue green aphids (*Acyrthosiphon kondoi*). In contrast, the susceptible genotype A17 could not trigger the JA signaling pathway to defend against blue green aphids (Gao *et al.*, 2008). In the current study, neither of the genotypes A17 or *sta-1* activated the JA signaling pathway when infested by pea aphid (Fig. 2). Thus, it appears that the JA signaling pathway in *M. truncatula* was not actually stimulated by pea aphid infestation under the well-watered conditions, even though it has been described as the most effective resistance factor against green peach aphid and blue green aphids (Ellis *et al.*, 2002; Gao *et al.*, 2008).

The up-regulation of the ABA signaling pathway under drought stress could affect the aphid penetration stages by altering the SA and JA signaling pathways. The expression of genes in the SA signaling pathway and the SA concentration of A17 plants were significantly decreased under drought stress, which allows aphids to spend less time to overcome the epidermis/mesophyll resistance of the plant (Fig. 2A, B). Furthermore, pea aphid-activated JA signaling in drought-stressed A17 plants consequently increased the mesophyll/phloem resistance of A17 plants as revealed by EPG data. When the ABA signaling pathway is defective, drought has little effect on the SA and JA signaling pathway in *sta-1*, which did not affect the aphid penetration stage. Furthermore, some proteins such as sieve element occlusion 1 (SEO1), SEO2, and SEO3 are located in the phloem and reported to prevent efficiently aphid feeding in *M. truncatula* (Will *et al.*, 2013). The SEO genes can be up-regulated by dehydration and artificial ABA treatment in the legume *Pisum sativum* (Srivastava *et al.*, 2014). Our results indicate that up-regulation of the JA signaling pathway and other defensive metabolites may increase the phloem defense during aphid penetration under drought conditions.

### Aphid phloem feeding stage

Once the aphid stylets reached the phloem, nutritional quality, especially N nutrition, would affect aphid feeding behavior because aphids prefer host plants with relatively high amino acid concentrations (Nowak and Komor, 2010). In the current study, drought stress decreased total leaf N concentration but did not affect the total amino acid concentration in plant leaves. The decreased N fixation ability under drought stress may contribute to the decreased N concentration of legume plants (Larrainzar *et al.*, 2007). Because feeding on the phloem sap in the plant sieve elements supports a substantial flux of non-essential amino acids, which the aphid endosymbiont *Buchnera* converts into essential amino acids (Hansen and Moran, 2011), we quantified the amino acid composition of both plant genotypes. The plant-stress hypothesis asserts that drought increases the hydroxylation of proteins, which subsequently increases the level of free amino acids that enhance insect growth and reproduction (White, 1974). However, in our current study, drought stress and genotype had little effect on the concentrations of individual amino acids except for proline (Fig. 6). Furthermore, proline acts as a mediator of osmotic adjustment and therefore may be acting here as a stress-related signal more than as a nutrition substrate for pea aphids in the plant (Szabados and Savouré, 2010). The increase in proline under drought stress does not appear to enhance the N nutrition available to aphids, and the up-regulated ABA signaling pathway has little effect on the individual amino acid concentration in the phloem of plants under drought stress.

### Aphid xylem absorption stage

Sustained aphid feeding on a host plant requires a relatively high plant water potential for two reasons. First, to feed on phloem, aphids require that the host cells maintain a high turgor pressure (Huberty and Denno, 2004). Secondly, aphids must absorb xylem sap to balance the osmotic pressure of the sugar-rich phloem sap and avoid dehydration (Daniels *et al.*, 2009; Nalam *et al.*, 2012). Previous studies found that aphids, like pathogens, can trigger stomatal closure, decrease leaf transpiration, and maintain the water content of the host plant by up-regulating the ABA signaling pathway. This manipulation of the host plant’s stomata helps aphids absorb water from the xylem to neutralize phloem osmotic pressure (Sun *et al.*, 2015). Furthermore, the SA signaling pathway was also reported to be involved in plant stomatal closure when challenged by biotic stress (Mateo *et al.*, 2004). We speculate that the aphid infestation-induced SA signaling pathway could partially decrease stomatal aperture and aid aphid xylem absorption. Aphid xylem absorption and osmoregulation could be affected by diverse environmental changes due to the changes of phytohormones and water status. For instance, elevated CO_2_ is beneficial to aphid xylem absorption and osmoregulation due to the increased water potential and water content of host plants (Sun *et al.*, 2015). We speculate that, under drought stress, a high water potential in the host plant is crucial for the aphid. In our current study, drought stress decreased the water potential and water content of both genotypes, which decreased xylem absorption by aphids. Activation of the ABA signaling pathway of A17 plants under drought stress increased water-use efficiency by decreasing stomatal aperture and transpiration. In contrast, the *sta-1* plants still maintained a higher transpiration rate, and their water-use efficiency and water potential were dramatically decreased when facing drought stress. Thus, we rarely detected any xylem absorption activities of pea aphids associated with *sta-1* plants on which the aphid has the lowest water content and highest osmolarity under drought stress. These results suggest that up-regulation of the ABA signaling pathway in A17 plants plays an important role in supporting xylem sap absorption by the aphid under drought stress.

In summary, although drought stress reduces epidermis/mesophyll resistance of *M. truncatula*, the performance of the pea aphid associated with wild-type plants was reduced by increasing host mesophyll/phloem resistance and by decreasing the host water status (Fig. 9). The feeding behavior and performance of aphids are more negatively affected by drought stress if the ABA signaling pathway of the host plant is deficient compared with the wild type. This study has generated several significant findings. First, our results show that drought significantly up-regulated the ABA signaling pathway, decreased plant epidermis/mesophyll resistance, and increased mesophyll/phloem resistance in the host plant, which prolonged the time required for the aphid stylets to reach the phloem. Secondly, drought stress decreased the plant N concentration, which did not depend on the ABA signaling pathway; these results are inconsistent with the view that drought stress increases the amino acid concentration and thereby enhances aphid growth and reproduction. Finally, and perhaps most importantly, up-regulation of the ABA signaling pathway increased water-use efficiency of A17 plants on which the pea aphid is able to absorb the xylem sap under drought conditions. When the ABA signaling pathway was deficient, the extreme water loss of *sta-1* plants under drought stress limited aphid xylem absorption and subsequently reduced phloem feeding and the increase in the population. Thus, the up-regulation of the ABA signaling pathway could improve the ability of pea aphids to overcome the partial negative effects from drought on aphid xylem absorption, and subsequently support normal osmoregulation and population abundance of the aphid.

## Supplementary data

Supplementary data are available at *JXB* online.

Supplementary Table S1 Primer sequences used for real-time quantitative PCR.

Supplementary Table S2. *F*- and *P*-values from MANOVAs for the effect of water treatments, *M. truncatula* genotypes, and pea aphid infestation on key metabolites and genes in the SA signaling pathway and JA signaling pathway of two *M. truncatula* genotypes.

Supplementary Table S3. *F*- and *P*-values from MANOVAs for the effect of water treatments, genotypes, and pea aphid infestation on stomatal conductance and water status of two *M. truncatula* genotypes.

Supplementary Table S4. *F*- and *P*-values from MANOVAs for the effect of water treatments and *M. truncatula* genotypes on hemolymph osmolarity and water content in pea aphids when feeding on two *M. truncatula* genotypes.

Supplementary Data
