# Supplementary material for: Up-regulation of abscisic acid signaling pathway facilitates aphid xylem absorption and osmoregulation under drought stress
Source: J Exp Bot. 2015 Nov 6;67(3):681–93. doi: 10.1093/jxb/erv481 (PMC4737068; doi:10.1093/jxb/erv481)
Supplement: Supplementary Data [file supp_67_3_681__index.html]

Up-regulation of abscisic acid signaling pathway facilitates aphid xylem absorption and osmoregulation under drought stress — Up-regulation of abscisic acid signaling pathway facilitates aphid xylem absorption and osmoregulation under drought stress — Supplementary Data 

# Up-regulation of abscisic acid signaling pathway facilitates aphid xylem absorption and osmoregulation under drought stress

## Supplementary Data

Data files

- Supplementary\_tables\_S1\_S4.pdf - Supplementary Data
