# Supplementary material for: Up-regulation of abscisic acid signaling pathway facilitates aphid xylem absorption and osmoregulation under drought stress
Source: J Exp Bot. 2015 Nov 6;67(3):681–93. doi: 10.1093/jxb/erv481 (PMC4737068; doi:10.1093/jxb/erv481)
Supplement: Supplementary Data [file supp_erv481_Supplementary_tables_S1_S4.pdf]

Table S1 Primer sequences used for real-time quantitative PCR.

Gene	Primer sequence（5’-3’）	Function
PR (*Pathogenesis-related protein*)	*F*: TTCATTTCCCATGTGAGTGC<br>*R*: GTCGTTACGCCTTTGGTTCG	*SA signaling pathway*
CHTN (*Endochitinase*)	*F:* CGTAGCCACCGACCCTGTTA<br>*R:* GCATTCAAGCCCTCCGTTTA	
BGL (*Endo-beta-1,3-glucanase*)	*F*: AGCCAATAATAGACTTCCTA<br>*R*: AACTTTCTCAAGAGCAGCAT	
OPR (*12-OPDA reductase*)	*F*: GAGGTTACGACCGAAATGAT<br>*R*: GTGTAGAATGTCGCCCTGTT	*JA signaling pathway*
PI (*Cysteine proteinase inhibitor*)	F: ACTCTTGAGGCTATTGATGC<br>R: GAAGTGGTAAATGATGGTGC	
ABR (*ABA-responsive protein*)	*F*: TGGAGGACCAGGAACCATTA<br>*R*: CCATCAGAGCCAGCCACAAT	*ABA signaling pathway*
β-actin	*F*: CAGCCCACTGGATGTCTGTA<br>*R*: GTAGCAGCGCAAATTGAAGA	*House keeping gene*

Table S2. *F* and *P* values from MANOVAs for the effect of water treatments, *M. truncatula* genotypes and pea aphid infestation on key metabolites and genes in SA signaling pathway and JA signaling pathway of two *M. truncatula* genotypes.

Measured indices	Value	Treatment$_{(df)}$						
		W[a]$_{(1,56)}$	Geno[b]$_{(1,56)}$	PA[c]$_{(1,56)}$	W×Geno$_{(1,56)}$	W×PA$_{(1,56)}$	Geno×PA$_{(1,56)}$	W×Geno×PA$_{(1,56)}$
ABA	*F*	22.3	48.6	4.29	8.99	0.429	1.62	3.86
	P	<0.001***	<0.001***	0.043*	0.004**	0.515	0.208	0.054
ABR	*F*	34.9	18.7	4.84	6.74	1.44	1.00	4.13
	P	<0.001***	<0.001***	0.032*	0.012*	0.235	0.321	0.047*
SA	*F*	6.92	10.5	17.8	4.84	0.663	4.38	4.62
	P	0.011*	0.002**	<0.001***	0.032*	0.419	0.041*	0.036*
PR1	*F*	4.52	13.5	24.7	5.83	1.27	4.72	6.58
	P	0.038	<0.001***	<0.001***	0.019*	0.265	0.034*	0.013*
BGL	*F*	7.32	14.8	21.6	4.25	0.232	5.23	6.30
	P	0.009	<0.001***	<0.001***	0.044*	0.632	0.026*	0.015*
CHTN	*F*	5.94	17.9	15.4	7.57	2.43	4.09	4.29
	P	0.018	<0.001***	<0.001***	0.008**	0.125	0.048*	0.043*

JA	*F*	6.44	51.7	4.01	5.23	3.20	4.52	4.57
	P	0.014*	<0.001***	0.050*	0.026*	0.079	0.038*	0.037*
OPDA	*F*	7.84	14.9	4.16	9.01	4.52	6.05	5.64
	P	0.007**	<0.001***	0.046*	0.004**	0.038*	0.017*	0.021*
OPR	*F*	4.47	34.6	5.64	23.4	4.05	4.62	4.33
	P	0.039*	<0.001***	0.021*	<0.001***	0.049*	0.036*	0.042*
PI	*F*	5.31	56.1	4.84	11.4	3.66	5.55	8.54
	P	0.025*	<0.001***	0.032*	0.001**	0.061	0.022*	0.005**

[a] Well-watered vs. Drought [b] Two genotypes of *M. truncatula* (A17 vs. *sta-1*). [c] Infected or non-infected with pea aphids. *<0.05, **<0.01, ***<0.001.

Table S3. *F* and *P* values from MANOVAs for the effect of water treatments, genotypes and pea aphid infestation on stomatal conductance and water status of two *M. truncatula* genotypes.

Measured indices	Value	Treatment$_{(df)}$						
		W[a] $_{(1,56)}$	Geno[b] $_{(1,56)}$	PA[c] $_{(1,56)}$	W×Geno$_{(1,56)}$	W×PA$_{(1,56)}$	Geno×PA$_{(1,56)}$	W×Geno×PA$_{(1,56)}$
g_s[d]	*F*	23.6	63.7	1.48	6.58	2.29	8.54	6.05
	P	<0.001***	<0.001***	0.234	0.013*	0.136	0.005**	0.017*
Tr[e]	*F*	67.7	21.8	3.98	9.01	2.21	9.01	4.38
	P	<0.001***	<0.001***	0.056	0.004**	0.143	0.004**	0.041
WEU[f]	*F*	34.6	47.9	3.32	7.32	2.94	6.44	6.91
	P	<0.001***	<0.001***	0.079	0.009**	0.092	0.014*	0.011*
WP[g]	*F*	27.6	68.1	3.04	2.46	0.626	4.84	5.64
	P	<0.001***	<0.001***	0.092	0.122	0.432	0.032*	0.021*
RWC[h]	*F*	18.0	124	1.46	3.44	0.303	4.16	3.78
	P	<0.001***	<0.001***	0.239	0.069	0.584	0.046*	0.057

[a] Well-watered vs. Drought. [b] Two genotypes of *M. truncatula* (A17 vs. *sta-1*). [c] Infected or non-infected with pea aphids. [d] stomatal conductance [e] transpiration rate [f] water-use efficiency. [g] water potential. [h] relative water content. *<0.05, **<0.01, ***<0.001.

Table S4. *F* and *P* values from MANOVAs for the effect of water treatments, *M. truncatula* genotypes on haemolymph osmolarity and water content in pea aphids when feeding on two *M. truncatula* genotypes.

Measured indices	Value	Treatment$_{(df)}$		
		W[a] $_{(1,28)}$	Geno[b] $_{(1,28)}$	W×Geno$_{(1,28)}$
HO[c]	*F*	45.2	13.4	0.302
	P	<0.001***	0.001**	0.587
WC[d]	*F*	30.5	11.6	0.160
	P	<0.001***	0.002**	0.692

[a] Well-watered vs. Drought. [b] Two genotypes of *M. truncatula* (A17 vs. *sta-1*). [c] haemolymph osmolarity . [d] water content. *<0.05, **<0.01, ***<0.001.
